# Supplementary material for: Association of Non-Invasive Positive Pressure Ventilation with Short-Term Clinical Outcomes in Patients Hospitalized for Acute Decompensated Heart Failure
Source: J Clin Med. 2021 Oct 29;10(21):5092. doi: 10.3390/jcm10215092 (PMC8584464; doi:10.3390/jcm10215092)
Supplement: Supplementary file 1 [file jcm-10-05092-s001.zip › jcm-1411249-supplementary.pdf]

Supplement

Results

The findings in the pre-matched cohort

In the pre-matched cohort, 775 (19.7%) patients received NPPV. Baseline characteristics of the NPPV and non-NPPV groups in the pre-matched cohort are presented in **Table S1**. The NPPV group exhibited a higher proportion of comorbidities such as atrial fibrillation, hypertension, dyslipidemia, and diabetes mellitus (all P<0.001), higher rate of admission in the third admission period (2014–2017, P<0.001), lower proportion of prior admission for ADHF (P<0.001), and lower percentage of medications prescribed for HF including β-blockers, mineralocorticoid receptor antagonists, and loop diuretics. The NPPV group had severe HF represented by lower oxygen saturation (SpO_2) (P<0.0001), higher heart rate (P<0.001), and higher B-type natriuretic peptide (BNP) levels (P<0.001) at the time of admission.

Despite comparable cardiac function parameters such as left ventricular ejection fraction (LVEF) or E/e′, a higher percentage of patients in the NPPV group received in-hospital treatment including vasodilators, loop diuretics, and catecholamines and mechanical supports including intra-aortic balloon pump. The NPPV group exhibited a higher ETI rate (**Figure S1A**), in-hospital mortality (**Figure S1B**), cardiac mortality (**Figure S1C**) and long er length of hospital stay (LOS, **Figure S1D**). The NPPV group also exhibited a higher rate of composite endpoints of all-cause mortality and ADHF rehospitalization within 90 days of discharge (P=0.021, **Figure S2**).

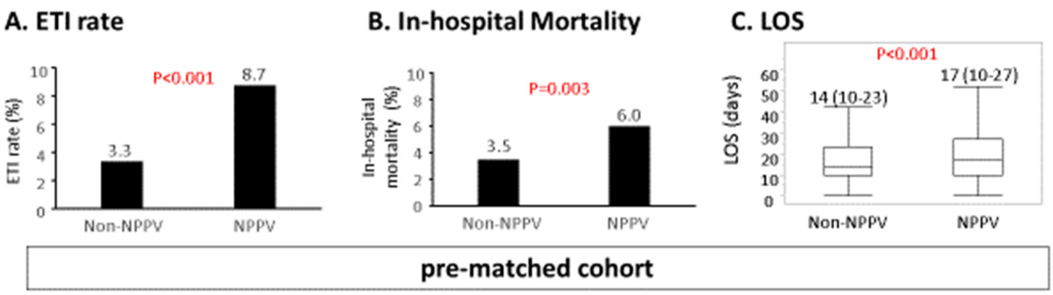

Figure S1. In-hospital outcomes in the pre-matched cohort. (A) ETI rate, (B) in-hospital mortality, and (C) LOS in the pre-matched cohort are depicted. ETI: endotracheal intubation, NPPV: non-invasive positive pressure ventilation, LOS: length of hospital stay.

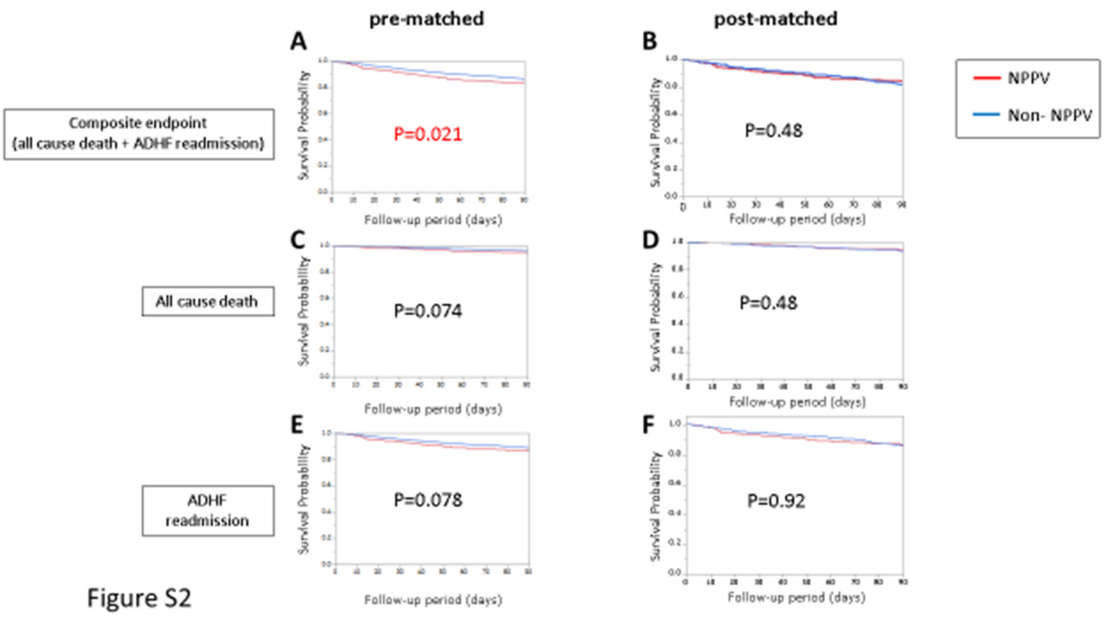

Figure S2. Short term survival analysis for 90 days after discharge. Shown are the Kaplan-Meier survival curves for the composite endpoint of all cause death and ADHF readmission (A and B), all cause death (C and D), and ADHF readmission (E and F) in the pre-matched and the post-matched cohort.

ADHF, acute decompensated heart failure; NPPV, non-invasive positive pressure ventilation.

Table S1. Baseline Characteristics According to Non-invasive Positive Pressure Ventilation Use in the pre-matched cohort

	pre-matched cohort			
	Non-NPPV (N=3152)	NPPV (N=775)	P Value	SDM
Year			<0.001	
2006-2009 (%)	493(16)	21(3)		0.46
2010-2013 (%)	1152(37)	281(36)		0.01
2014-2017 (%)	1507 (48)	473(61)		0.27
Age (years)	77 (67- 84)	78 (68 - 84)	0.070	0.08
Female (%)	1251 (40)	325 (42)	0.25	0.04
BMI	22.9 (20.4 – 25.6)	23.0 (20.3 – 25.8)	0.90	0.001
Etiology			<0.001	
DCM (%)	483 (15)	64 (8)		0.27
ICM (%)	840 (27)	302 (39)		0.26
VHD (%)	856 (27)	184 (24)		0.08
HFr/mr/pEF			0.019	
HFrEF (%)	1256(40)	315(41)		0.02
HFmrEF (%)	533(17)	158(21)		0.09
HFpEF (%)	1322(42)	287(38)		0.095
Prior ADHF admission (%)	992 (32)	189 (24)	<0.001	0.16
Atrial Fibrillation (%)	1576 (50)	289 (37)	<0.001	0.26
Hypertension (%)	1988 (63)	592 (76)	<0.001	0.29
Dyslipidemia (%)	1147 (37)	349 (45)	<0.001	0.17
DM (%)	1039 (33)	311 (40)	<0.001	0.15
Smoking (%)	1280 (42)	336 (44)	0.27	0.04
Dialysis (%)	85 (3)	50 (6)	<0.001	0.18
COPD (%)	155 (5)	39 (5)	0.90	0.01
Stroke/TIA (%)	424 (14)	116 (15)	0.29	0.03
Home oxygen therapy (%)	91 (3)	37 (5)	0.012	0.1
Pacemaker (%)	257 (8)	36 (5)	<0.001	0.14
ICD (%)	129 (4)	16 (2)	<0.001	0.12
CRT (%)	57 (2)	5 (1)	0.01	0.1
**Clinical Presentation at Admission**				
NYHA II/III/IV			<0.001	
II (%)	637 (21)	80 (11)		0.30
III (%)	1212 (41)	201 (26)		0.31
IV (%)	1128 (38)	481 (63)		0.52

SBP (mmHg)	134 (115 – 154)	150 (125 – 181)	<0.001	0.52
Heart rate (bpm)	88 (71 – 108)	103 (82 – 121)	<0.001	0.42
SpO2 (%)	96 (94 – 98)	93 (88 – 97)	<0.001	0.57
PND (%)	1001 (34)	339 (48)	<0.001	0.29
Orthopnea (%)	1111 (40)	395 (65)	<0.001	0.52
Rales (%)	1401 (47)	496 (70)	<0.001	0.47
Sound III (%)	1076 (36)	302 (42)	0.002	0.13
JVD (%)	1308 (47)	307 (50)	0.16	0.06
Edema (%)	2076 (68)	454 (62)	0.002	0.12
Cold extremities (%)	472 (18)	224 (37)	<0.001	0.45
**Laboratory Data**				
BNP/NT-proBNP quartile			<0.001	
1st	803(26)	135(18)		0.20
2nd	774 (26)	173 (23)		0.06
3rd	760(25)	191(26)		0.01
4th	696(23)	249(33)		0.23
BNP (pg/ml)	697 (356 – 1234)	901 (471 – 1588)	<0.001	0.25
NT-pBNP (pg/ml)	3658 (1857 – 7863)	4922 (2595 – 12074)	<0.001	0.22
Hemoglobin (g/dl)	12.0 (10.4 – 13.7)	11.8 (10.3 – 13.7)	0.35	0.03
BUN (mg/dl)	22 (16.5 – 31.3)	22.8 (17 – 35.1)	0.018	0.12
eGFR (ml/min/1.73m2)	49.8 (34.2 – 64.5)	46.6 (28.8 – 63.1)	<0.001	0.11
UA (mg/dl)	6.6 (5.4-8.1)	6.4 (5.3-8.0)	0.12	0.08
Na (mEq/l)	140 (137 – 142)	139 (137 – 142)	<0.001	0.16
CRP (mg/dl)	0.48 (0.14 – 1.52)	0.72 (0.19 – 2.86)	<0.001	0.26
**Echocardiography**				
LVDd (mm)	52 (45 – 59)	51 (45 – 57)	0.13	0.07
LVEF (%)	45 (31 – 59)	43 (32 – 57)	0.38	0.04
LAD (mm)	44 (39 – 50)	42 (37 – 47)	<0.001	0.29
E/e′	17.0 (11.7-24.8)	15.8 (9.1-24.0)	0.003	0.11
TRPG (mmHg)	30 (23-38)	28 (22-36)	0.032	0.11
**Pre-hospital treatment**				
ACE-I/ARB (%)	1325 (42)	348 (45)	0.14	0.06
β-blocker (%)	1420 (45)	295 (38)	<0.001	0.14
MRA (%)	524 (17)	80 (10)	<0.001	0.2
Loop diuretic(po.) (%)	1485 (47)	279 (36)	<0.001	0.23
Thiazide (%)	170 (6)	34 (5)	0.25	0.05
**In-hospital treatment**				
Loop diuretics（iv.）(%)	2002 (65)	598 (77)	<0.001	0.27
Nitrates (%)	634 (20)	402 (52)	<0.001	0.7
Carperitide (%)	1444 (46)	452 (58)	<0.001	0.25
PDE-III (%)	81 (3)	29 (4)	0.09	0.07
Catecholamine(%)	371 (12)	197 (25)	<0.001	0.35
IABP (%)	44 (1)	34 (4)	<0.001	0.18

Categorical values are expressed as percentage, and continuous variables are expressed as

median (interquartile range). NPPV, non-invasive positive pressure ventilation; SDM, standardized difference in means; BMI, body mass index; DCM, dilated cardiomyopathy; ICM, ischemic cardiomyopathy; VHD, valvular heart disease; HFrEF, HF with reduced ejection fraction; HFmrEF, HF with mid-range ejection fraction; HFpEF, HF with preserved ejection fraction; NYHA, New York Heart Association functional class; ADHF, acute decompensated heart failure; DM, diabetes mellitus; COPD, chronic obstructive pulmonary disease; TIA, transient ischemic attacks; HOT, home oxygen therapy; ICD, implantable cardioverter defibrillator; CRT, cardiac resynchronization therapy; SBP, systolic blood pressure; SpO2, oxygen saturation; PND, paroxysmal nocturnal dyspnea; JVD, Jugular venous distention; BNP, B-type natriuretic peptide; NT-pro BNP, N-terminal pro B-type natriuretic peptide; BUN, blood urea nitrogen; Cr, serum creatinine; eGFR, estimated glomerular filtration rate; UA uric acid; Na, serum sodium; T Bil, total bilirubin; CRP, C-reactive protein; LVDd, left ventricular end-diastolic diameter; LVDs, left ventricular end-systolic diameter; LAD, left atrial diameter; TRPG, tricuspid regurgitation pressure gradient; ACEI, angiotensin-converting enzyme inhibitor; ARB, angiotensin receptor blocker; MRA, mineralocorticoid antagonist; PDE-III, phosphodiesterase-III inhibitor; IABP intra-aortic balloon pumping.
